# Zinc-based amendments and Zn-solubilizing beneficial bacteria mitigate lead-induced toxicity in chickpea (*Cicer arietinum* L.)

**DOI:** 10.3389/fmicb.2025.1718122

**Published:** 2025-12-12

**Authors:** Mohammad Shahid, Mohammad Abul Farah, Khalid Mashay Al-Anazi, Leonard Koolman

**Affiliations:** 1 Marwadi University Research Center, Department of Agriculture, Faculty of Science, Marwadi University, Rajkot, Gujrat, India; 2 Department of Zoology, College of Science, King Saud University, Riyadh, Saudi Arabia; 3 Centre for sAMR and One Health Research, School of Biological, Health and Sports Science, Technological University Dublin, Dublin, Ireland

**Keywords:** *Cicer arietinum* (L.), *E. cloacae*, Lead (pb)-detoxification, Zn and ZnO-NPs, reduced ROS accumulation, bacterial-assisted nano-phytoremediation

## Abstract

Lead (Pb) is a toxic metal that disrupts plant physiological processes and poses significant human health risks. Fertilizers, nanomaterials, and mineral-solubilizing plant growth-promoting rhizobacteria (PGPR) support phytoremediation as an emerging, eco-friendly strategy for metal detoxification by improving nutrient availability and minimizing metal toxicity in plants. This study explored the interactions of Zn-fertilizer, zinc-oxide nanoparticles (ZnO-NPs), and Zn-solubilizing *Enterobacter cloacae* (Zns-*Ec*) in mitigating Pb-induced phytotoxicity in 50, 100, and 150 mgPbkg^−1^ soil-stressed chickpea. Under Pb stress. Zn, ZnO-NP and Zns-*Ec* significantly (*p* < 0.05) improved root biomass (23.4, 52.3, and 71.4%), chl *a* (21.2, 26.5, and 39.8%), and carotenoids (26.7, 29.7 and 45.7%) in chickpea subjected to 50 mgPbkg^−1^ soil. Zn, ZnO-NP and Zns-*Ec* interactions reduced lipid peroxidation (LPO), hydrogen peroxide (H_2_O_2_) and membrane integrity and increased soluble proline, helping to mitigate oxidative stress in Pb-stressed chickpea. Zn, ZnO-NP and Zns-*Ec* increased antioxidant enzyme activity of chickpea with highest increases in catalase (CAT; 33.4, 40.7 and 51.2%), ascorbate peroxidase (APX; 24.5, 29.8, and 41.2%), glutathione reductase (GR; 20.7, 33.1 and 48.9%), peroxidase (POD; 24.8, 34.5, and 48.9%) under 150 mgPbkg^−1^ stress. Treatments of Zn, ZnO-NP and Zns-*Ec* greatly reduced Pb uptake in roots (20.1, 35.7, and 67%) and shoots (17.6, 29.5 and 54%), in 50 mgPbkg^−1^-stressed chickpeas. Additionally, Zn amendments significantly reduced Pb concentrations in rhizosphere soil, increased levels of available K and soluble P. Rhizosphere soil harvested from Zn, ZnO-NPs, and Zns-*Ec* treatments showed increased available P (8.2 24.5 and 29.5%) and K (24, 21, 34.5%) content under mgPbkg^−1^ Pb treatment. Zn, ZnO-NP, and Zns-*Ec* mitigated Pb toxicity in chickpea by enhancing antioxidant enzyme activity, reducing lipid peroxidation, and limiting Pb uptake through Zn–Pb interaction and rhizosphere immobilization. These findings the synergistic role of Zn amendments and metal-tolerant PGPR in improving plant resilience under heavy metal stress.

## 1 Introduction

Plants worldwide face threats from a range of heavy metals (HMs), which endangers their health and survival (Acharya, 2024; Asiminicesei et al., 2024). Among these, lead (Pb) is one of the most persistent and widespread toxic metals in agricultural soils, posing a serious global concern. Elevated Pb levels have been reported from farmlands across Asia, Africa, and Europe, where industrial and agricultural activities have accelerated soil contamination (Singh et al., 2024). The accumulation of Pb in edible crops represents a major threat to food safety and human health, particularly in developing nations that rely heavily on contaminated soils for cultivation (Ali et al., 2024). The Pb contamination in soils ranges from 400 to 800 mgkg^−1^, with industrial areas reaching up to 1,000 mgkg^−1^. Lead contamination adversely affects soil biological activity, reducing crop productivity (Alengebawy et al., 2021). Additionally, Pb disrupts ecosystems and biodiversity by affecting soil organisms, plants, and domestic and wild animals (Collin et al., 2022).

Legumes, particularly chickpea (*Cicer arietinum* L.), play a vital role in global food and nutritional security by providing protein-rich grains and improving soil fertility through nitrogen fixation (Mahto et al., 2025). However, chickpea is highly sensitive to heavy metals, especially Pb, which reduces yield, nodulation efficiency, and nutrient assimilation (Wani et al., 2007). Hence, mitigating Pb toxicity in legumes is essential for sustaining productivity under polluted conditions. In 2020, chickpea was cultivated on 14.5 million hectares across 56 countries, producing about 15.1 million tonnes (Kumar et al., 2025), underscoring its global importance. Pb uptake, transport, and distribution in plants depend on its soil speciation, cation exchange capacity, particle size, pH, and plant genotype (Rahman et al., 2024; Gupta M. et al., 2024: Gupta S. et al., 2024). Excessive Pb disrupts mineral nutrition, photosynthesis, and hormonal balance, leading to oxidative stress, membrane damage, and ultimately cell death (Gupta M. et al., 2024; Gupta S. et al., 2024; Jagota et al., 2024).

Mitigating Pb toxicity is thus essential for sustaining legume productivity and food safety. Nutrient management offers a cost-effective and eco-friendly strategy to alleviate HM toxicity, as adequate supply of essential elements supports plant metabolism and stress tolerance (Aziz et al., 2023; Ingestad and Ågren, 1995). Among micronutrients, zinc (Zn) plays a crucial role in reducing metal accumulation and oxidative stress in plants (Hassan et al., 2022). Zinc application, through soil or foliar routes, has been shown to limit HM uptake and translocation (Beltrame et al., 2025); however, its role in mitigating Pb accumulation in chickpea remains underexplored.

Nano-remediation with specific nanoparticles (NPs) is viable substitute for destroying or sequestering agricultural contaminants (Karnwal et al., 2024). Researchers have demonstrated that NPs enhance plant growth by reducing metal phytotoxicity (Chowdhury et al., 2025; Deng et al., 2024). Because they leave fewer environmental residues than traditional fertilizers, nano-fertilizers offer a promising substitute (El-Saadony et al., 2021). With the potential to increase growth and yield to meet the demands of a growing global population, ZnO-NPs hold promise for improving agricultural productivity (Jafir et al., 2024; Khan et al., 2023). Micronutrient-based NPs have been found to improve plant growth, antioxidative capacity, ionic balance, and seed germination, and mitigate metal toxicity (Qin et al., 2024; Nazir et al., 2022). Micronutrient nano-fertilizers help detoxify HMs for sustainable agriculture by increasing soybean growth and decreasing Cr accumulation (Basit et al., 2023).

Metal-tolerant plant growth-promoting rhizobacteria (MT-PGPR) are beneficial soil bacterial capable of enhancing plant growth while tolerating heavy metal stress (Oubohssaine et al., 2022). They applied in conjunction with phytoremediation can also be applied to address Pb-induced phytotoxicity (Ahmad et al., 2018). By promoting plant growth through production of PGPR compounds and nutrient uptake, microbe-assisted phytoremediation efficiently cleans up metal-polluted soils (Yu et al., 2024). PGPR have been documented to enhance plant growth under Pb stress and decrease HM toxicity (Madhogaria et al., 2024). Certain PGPR generate organic acids (OA) which increase HM bioavailability and aid in metal phytoextraction (Qin et al., 2024). Through processes like metal ion exclusion, intracellular accumulation, biotransformation to fewer toxic forms, and adsorption/desorption, MT bacteria are capable of withstanding metal stress (Gupta M. et al., 2024; Gupta S. et al., 2024). Rhizosphere microbes employ a variety of processes, including production of siderophores and acids as well as dissolution of phosphate (P) to promote HM transfer and mobilization (Han et al., 2024). Also, they can enhance the phytoremediation by producing plant growth regulators (PGRs) like auxins, gibberellins, and cytokinin, mimicking the effects of exogenous growth regulators (Arshad et al., 2023). MT-PGPR also promote plant growth by reducing ethylene-induced stress through the synthesis of ACC deaminase (Kour et al., 2024). Microbes interact with plants to produce signalling chemicals, promoting biofilm formation on the root surface and triggering a defense response (Mhlongo et al., 2018). The combined effect of micronutrients, NPs and MT-PGPR on reducing Pb toxicity in plants is underexplored, highlighting the need to assess their interactions on metal accumulation in edible crops.

Despite advances in nano- and microbe-assisted remediation, the tripartite interaction among nanoparticles, metal-tolerant rhizobacteria, and plants remains poorly understood, particularly regarding how these systems jointly influence Zn biofortification and Pb detoxification in legumes. Existing studies have explored either nutrient amendments or microbial inoculants individually, but their combined potential for improving Zn bioavailability, nutrient homeostasis, and antioxidant defense under Pb stress has not been fully elucidated. We hypothesized that the synergistic use of ZnO-NPs and ZnS *E. cloacae* MD-68 enhances Zn uptake and alleviates Pb-induced toxicity in chickpea by strengthening antioxidant defense and maintaining nutrient balance.

This study applied Zn, ZnO-NPs and a Zn- solubilizing PGPR strain to investigate their effect on chickpea growth in soil contaminated with 50, 100 and 150 mgPbkg^−1^. The primary objectives of this study were to investigate: (i) the effects of Zn, ZnO-NPs and zinc solubilizing *E. cloacae* (Zns-*Ec*- MD-68) on growth, biomass yield, and leaf pigments in chickpea plants under Pb toxicity; (ii) oxidative stress and the response of antioxidant enzymes in Pb-exposed and treated plants; and (iii) essential nutrient levels and metal uptake in chickpea plants in the presence of Pb, Zn, ZnO-NPs and a Zns-*Ec* strain. Through these assessments, we aimed to better understand how Zn, ZnO-NPs and MT-PGPR alleviate Pb-induced stress in chickpea.

## 2 Materials and methods

### 2.1 Isolation of zinc-solubilizing (ZnS) bacteria

Samples of rhizosphere soil were collected from the root zone of robust plants, allowed to air-dry (over-night, at room temperature), and then serially diluted to 10^−6^. Zinc-solubilizing agar medium (ZSAM) containing insoluble zinc carbonate (ZnCO₃) as the only zinc source was dosed with aliquots (0.1 mL) of the appropriate dilution (Hashemnejad et al., 2021). Plates were incubated at 28 ± 2 °C for 3–5 d. Those bacterial colonies that formed a clear halo (i.e., zone of solubilization) were designated as Zn solubilizers. The following formula was used to determine Solubilization Index (SI):

.Solubilization index(SI)=Halo zone diameter/Colony diameter

Representative colonies were purified by continuous streaking (2–3 times), maintained on nutrient agar (NA) slants at 4 °C, and preserved in glycerol stocks (−80 °C) for further characterization.

### 2.2 Estimation of Zn solubilizing ability based on qualitative assay

The zinc-solubilizing (ZnS) bacterial isolates were spot-inoculated (10 μL) onto NB agar supplemented with insoluble zinc compounds and incubated at 28 °C for up to 72 h. At 48 and 72 h, development of clear halo zones surrounding colonies was observed. The zinc solubilization index (ZSI) and zinc solubilization efficiency (ZSE) were computed as follows (Gontia-Mishra et al., 2017).


ZSE=Halo zone diameter(mm)/Colony diameter(mm).

$$\begin{array}{l} \mathrm{ZSI}=\mathrm{Halo zone diameter}\;\left(\mathrm{mm}\right)-\mathrm{Colony diameter}\;\left(\mathrm{mm}\right)/\\ \mathrm{Colony diameter}\;\left(\mathrm{mm}\right). \end{array}$$

### 2.3 Quantitative evaluation of Zn solubilization

Following qualitative screening, a 1 mL suspension of actively growing ZnS-bacterial isolates was added to 20 mL of modified nutrient broth (NB; gmL^−1^: dextrose 10; (NH₄)₂SO₄ 1.0; KCl 0.2; K₂HPO₄ 0.1; MgSO₄ 0.1 ZnO 0.1; pH 7.0) The mixture was incubated at 28 ± 1 °C for 7 d under continuous shaking (120 rpm) in a completely randomized design at benchtop with three replications. Following incubation, cultures were centrifuged for 15 min at 8000 rpm. The supernatant was filtered and analyzed by atomic absorption spectrophotometry (AAS) to determine quantity of soluble Zn. The difference between Zn concentration in inoculated samples and uninoculated controls was used to compute the net solubilized Zn (μg mL^−1^).

### 2.4 Growth-promoting activities of rhizobacteria

Plant growth-promoting substances including indole-3-acetic acid (IAA) (Bric et al., 1991), exopolysaccharides (EPS) (Upadhyay et al., 2011), ammonia (Bladen et al., 1961) and siderophores (Reeves et al., 1983) released/synthesized by recovered rhizobacteria were evaluated. For detailed methodology, see Supplementary information.

### 2.5 Lead (pb) tolerance assay of recovered rhizobacteria

A plate assay was used to evaluate the resistance of bacterial isolates to lead (Pb). Nutrient agar (NA) medium dosed with increasing concentrations (50, 100, 200, 400, 600, 800, and 1,000 mg mL^−1^) of lead nitrate [Pb(NO₃)₂] was prepared. To the Pb-amended plates, 10 μL of actively growing bacterial cultures (24 h old) were spot-inoculated, and plates were incubated for 48–72 h at 28 ± 2 °C. The maximum tolerable concentration (MTC) of Pb was identified as the highest concentration at which visible growth was observed after bacterial growth was visually documented (Nath et al., 2018).

### 2.6 Molecular identification

Following production of growth-promoting substances and metal tolerance assessment, the *E. cloacae* MD-68 possessing strong PGP traits and high Pb tolerance levels, was identified at species level using 16S rRNA sequencing. See Supplementary information for genomic DNA extraction, PCR and sequencing.

### 2.7 Effect of Pb on PGP traits of *E. cloacae*

In order to assess the effect of Pb concentration (50, 100, 150, and 200 mg mL^−1^) on growth- promoting traits of *E. cloacae* MD-68, cells were cultured in the respective media, each dosed with the desired Pb concentration. PGP traits including production of IAA, EPS, ammonia, Zn and siderophores were determined using standard protocols as described by Danish et al. (2025) and Shahid et al. (2025).

### 2.8 Assessing tripartite interaction: mitigation of pb-induced toxicity in chickpea using Zn fertilizer, ZnO-NPs and ZnS-*E. cloacae* MD-68

#### 2.8.1 Biosafety and ethical considerations

All experiments involving Pb and microbial cultures were conducted following standard laboratory safety guidelines and institutional biosafety protocols. Appropriate personal protective equipment (PPE) was used at all times, and all chemical and microbial waste was disposed of according to environmental and biosafety regulations. No ethical approval was required as the study did not involve human or animal subjects.

#### 2.8.2 Soil collection, preparation, plant material, and treatment

The experiment was conducted using a sandy loam soil (60% sand, 25% silt, and 15% clay) collected from the agricultural fields. The soil was allowed to air-dry and then sieved to pass through a 2-mm mesh sieve. The soil had a pH range of 6.5–7 and low available Zn content (< 1 mg kg^−1^ DTPA-extractable). Each plastic pot (5.0 kg capacity) was filled with 4.0 kg soil, with drainage holes lined using a thin inert mesh. The experiment was conducted in a factorial arrangement to assess the effects of three lead (Pb) levels (50, 100, and 150 mg Pb kg^−1^ soil) and four amelioration treatments: water spray (T1 = control), foliar-applied Zn (T2), foliar-applied ZnO-NPs (T3), and Zn-solubilizing *E. cloacae* MD-68 (T4) (Supplementary Table S1). Each treatment combination was replicated in three pots (*n* = 3), with one pot serving as a single experimental unit and 3–4 plants maintained per pot after thinning. The study was laid out in a completely randomized design (CRD) with three replicates per treatment combination. Each pot was considered a single experimental unit, and 3–4 plants were maintained per pot after thinning. Plants were grown for up to 50–60 d after sowing (DAS) for physiological and biochemical measurements, and extended to maturity when yield attributes were assessed. Lead treatments were imposed using analytical grade lead nitrate [Pb(NO₃)₂] as the Pb source. Seeds of chickpea were surface-sterilized (70% ethanol, 30 s; 2% NaOCl, 2–3 min; rinsed 5 × sterile distilled water). The Zns-*Ec* MD-68 was cultured in NB to late log phase. For seed coating, bacterial suspensions (10^8^ CFU mL^−1^ in 1% CMC) were applied to deliver ~10^6^–10^7^ CFU seed^−1^, and coated seeds were shade-dried for 30 min. A soil drench (10 mL pot^−1^; 10^8^ CFU mL^−1^) was applied at sowing and again at 21 DAS. Seeds were sown to a 2–3 cm depth (3–4 seeds pot^−1^) and thinned at 10–12 DAS to 2–3 uniform plants. Soil moisture was maintained at 60–70%. Plants were grown under greenhouse conditions (24–28 °C Day/ 18–22 °C night, natural light). Pest and disease management was via non-metal formulations; Zn sprays were avoided in non-Zn treatments. The ZnSO_4_ treatment included 0.5% zinc sulphate (ZnSO₄·7H₂O) + 0.25% Ca(OH)₂ + 0.05% Tween-20, and was applied twice (25–30 DAS, 45 DAS) to incipient runoff (~25–35 mL plant^−1^) with a fine hollow-cone nozzle each morning. The ZnO nanoparticle (Zn-NPs) treatment was as follows: ZnO-NPs (20–50 nm) at 50 mg mL^−1^ in deionized water + 0.05% Tween-20, sonicated 10–15 min before spraying. Sprays were given at the same timings and volume as ZnSO₄. The control received distilled water.

Data were statistically analyzed using SPSS 16.0 for Windows (Illinois, USA). Differences among treatment means were determined by one-way ANOVA, followed by Duncan’s multiple range test (DMRT) at a 95% confidence level (*p* < 0.05). Pearson correlation analysis was performed to evaluate relationships among physiological and biochemical parameters, and principal component analysis (PCA) and heat map visualization were conducted using Origin software to assess treatment interactions.

#### 2.8.3 Measurement of root and shoot length and number of branches

Three plants were carefully removed from each pot for each treatment. A measuring tape or scale was used to measure the lengths of the roots and shoots, and the number of primary branches per plant was recorded.

#### 2.8.4 Measurement of fresh and dry biomass

Plants were carefully uprooted, washed free of soil particles using tap water, and separated into shoot and root sections. The fresh mass (FM) of each section was recorded immediately. The dry mass (DM) of the samples was determined after oven drying for 48 h at 80 °C. The dry-to-fresh mass ratio (%) for each section was calculated using a slightly modified formula described by Kausar et al. (2012).

#### 2.8.5 Estimation of leaf pigments (chlorophyll and carotenoid) in chickpea

To estimate the chlorophyll and carotenoids contents in fresh foliage of Pb-treated, Zn, ZnO-NPs and Zns-*Ec* applied chickpea, leaf samples (500 mg) were ground in chilled acetone (80%) using an agate mortar and pestle. The absorbance of the supernatant was recorded at 663 nm, 645 nm, and 470 nm for chl a, chl b, and carotenoid content, respectively, using a UV–vis spectrophotometer (UV-1900, Shimadzu, Japan). The calculations of the pigment concentrations were performed as outlined by Arnon (1949) and Kirk and Allen (1965).

#### 2.8.6 Chlorophyll fluorescence measurements

A pulse-amplitude modulated fluorometer (PAM, Heinz Walz, Germany) was used to measure chlorophyll fluorescence on fully expanded chickpea leaves from each treatment (Pb, Pb + Zn fertilizer, ZnO-NPs, Zns-*Ec*, and Control) (Danish et al., 2024). To determine maximum PSII efficiency, leaves were dark-adapted for 20 min using leaf clips. Minimal (F₀) and maximal (Fₘ) fluorescence were subsequently measured:


$$F_{v}/F_{m}=\left(F_{m}-F_{0}\right)/F_{m}.$$

Plants were then subjected to actinic light (300–500 μmol m^−2^ s^−1^) in order to achieve both light-adapted maximum fluorescence (*F*ₘ′) and steady-state fluorescence (*F*ₛ). The PSII effective quantum yield was calculated from:


$$\Phi \mathrm{PSII}=\left(F_{m}\prime -F_{s}\right)/F_{m}\prime .$$

Photochemical quenching (qP) was calculated from:


$$\mathrm{qP}=\left(F_{m}\prime -F_{s}\right)/\left(F_{m}\prime -F_{0}\prime \right).$$

where F₀′ was obtained under far-red illumination.

#### 2.8.7 Gas exchange attributes of pb-stressed chickpea under Zn, ZnO-NPs and Zns-*Ec*

The fully expanded chickpea leaves were detached from each treatment (Pb, Zn, ZnO-NPs and Zns-*Ec*-inoculated) between 9:00 and 11:00 a.m. under ambient CO₂, light, and temperature conditions. Gas-exchange parameters such as stomatal conductance (gs), net photosynthetic rate (Pn), intercellular CO₂ concentration (Ci), and intrinsic water use efficiency (iWUE = Pn/gs) were measured using a portable photosynthesis system (e.g., LI-6400XT, LI-COR, USA). Leaf water potential (Ψw, %) was measured immediately following gas-exchange analysis (Holá et al., 2010).

#### 2.8.8 Relative water content

The relative leaf water content (RWC) in metal-treated, Zn fertilizer, ZnO-NP and Zns-*Ec* inoculated chickpea plants was evaluated using the equation given by Barrs and Weatherley (1962).


RWC(%)=(FM−DM)/(TM−DM)×100

where FM represents fresh mass, TM denotes turgid mass, and DM indicates dry mass.

### 2.9 Pb-indued oxidative stress parameters in Zn, ZnO-NP and Zns-*Ec* -treated chickpea

#### 2.9.1 Electrolyte leakage (EL)

Electrolyte leakage (EL) in Pb-treated and Zn, ZnO-NP and Zns-*Ec* -applied chickpea plants was assessed using the method of Lutts et al. (1996) with slight modifications. Fresh leaf discs were excised and dipped in a test tube containing 25 mL of distilled water (ddH_2_O), and left overnight at room temperature. Conductivity (EC1) was recorded at 25 °C using a conductivity meter. Another set of tubes containing the cut leaf discs was kept in a water bath for 20 min and the final electrical conductivity (EC2) was noted.


Electrolyte leakage(EL%)=(EC1/EC2)×100.

#### 2.9.2 Malondialdehyde (MDA)

Leaf samples (0.5 g) were crushed in 2.5 mL of 5% thio-barbituric acid (TBA) and the suspension diluted with 5% trichloroacetic acid (TCA). The finely crushed samples were heated at 90 °C for 20 min in water bath following which they were promptly chilled on ice. Absorbance was determined at 532 and 600 nm employing a UV–vis spectrophotometer (Heath and Packer, 1968).

#### 2.9.3 Hydrogen peroxide (H_2_O_2_)

Plant tissue (0.5 g root samples) was homogenized using mortar and pestle in phosphate buffer and then centrifuged at 6000 × *g*. The supernatant was mixed with a solution of 0.1% titanium sulfate and 20% (v/v) H_2_SO_4_. At 410 nm, the calorimetric intensity of the yellow color was measured (Kwasniewski et al., 2013). Using known H_2_O_2_ concentrations, a standard curve was constructed to determine the H_2_O_2_ values. The tissue sample reaction mixture served as a control.

#### 2.9.4 Proline content

Proline content was determined in Pb-treated, Zn fertilizer, ZnO-NP and Zns-*Ec* inoculated chickpea plants using the method of Bates et al. (1973).

### 2.10 Antioxidant enzyme activity

Fresh leaves (0.3 g) were homogenized in 2.0 mL of 50 mM phosphate buffer (pH 7.8) containing 0.2 mM EDTA and centrifuged at 13,000 rpm for 25 min at 4 °C. The supernatant was used to estimate soluble protein and antioxidant enzyme activities. Catalase (CAT) activity was measured by the decline in H₂O₂ absorbance at 240 nm (Cakmak and Marschner, 1992). Ascorbate peroxidase (APX) was assayed by monitoring ascorbate oxidation at 290 nm (Nakano and Asada, 1981). Guaiacol peroxidase (GPX) activity was determined using o-phenylenediamine as a chromogenic substrate, recording absorbance at 417 nm (Vetter et al., 1958). Superoxide dismutase (SOD; EC 1.15.1.1) was assayed by the inhibition of NBT reduction at 560 nm (Dhindsa et al., 1982). Glutathione reductase (GR) activity was determined from the decline in NADPH absorbance at 340 nm (Yang et al., 2006). Enzyme activities were expressed as units per mg protein.

### 2.11 Analysis of metal concentrations in plant tissue

Harvested roots and shoots from each treatment were carefully rinsed with ddH_2_O, allowed to air-dry at room temperature, and then oven-dried for 12 h at 60 °C. Dried samples were ground to a fine powder using a mesh grinder. A total of 0.5 g of each sample was digested at 100–230 °C in digestion tubes containing 4.0 mL HNO₃ and 1.0 mL HClO₄. A clear solution was obtained by passing digests through a 0.45 μm membrane filter. Concentrations were measured using flame atomic absorption spectrophotometry (Perkin Elmer 700) (Zafar et al., 2010).

### 2.12 Concentration of zinc in plant samples

One gram of dried and ground plant material was digested in a solution of HClO₄ and HNO₃ (4,1, v/v). The mixture was pre-digested for one night at room temperature, following which it was diGomesgested on a hot plate at 150–250 °C until the solution turned clear. Digests were cooled, filtered, and made to volume (100 mL) using deionized water. Zinc concentration of the digests was measured using atomic absorption spectrophotometry (Yoshida et al., 1976).

### 2.13 Analysis of soil samples

Soil pH was determined on a mixture of 1.0 g soil in 10 mL H_2_O using a glass electrode pH meter. Available phosphorus (AP) was measured using molybdenum–antimony colorimetry (OD₇₀₀). One gram of soil was mixed with 7 mL of ammonium fluoride solution, shaken for 1 min, and filtered (Whatman no. 1 filter paper). Available potassium (AK) was determined by mixing 5 g of soil that had been sieved through a 20-mesh screen, with 50 mL of neutral ammonium acetate. The mixture was shaken for 30 min and filtered through Whatman no. 1.0 filter paper. The AK was determined in the filtrate using a flame photometer. Total lead (Pb) concentration was determined via microwave digestion. A total of 5.0 g soil was mixed with 10 mL nitric acid and 0.5 mL hydrofluoric acid. The mix was digested at 180 °C for 90 min to produce a clear solution. Using xylenol orange spectrophotometry, Pb concentration of the digests was measured.

### 2.14 Statistical analysis and multivariate analysis

Statistical analysis was conducted using SPSS 16.0 for Windows (Illinois, USA). All values were expressed as mean ± standard error. Means were compared using analysis of variance (ANOVA), and comparisons of means were performed using the DMRT test. Significance was estimated at a confidence level of 95% (*p* < 0.05%). Pearson, heat map and PCA statistical tests were performed to test correlations between plant physio-biochemical parameters.

## 3 Results and discussion

### 3.1 Isolation of Zn-solubilizing rhizobacteria

A total of twelve Zn-solubilizing (ZnS) rhizobacterial isolates were recovered from the chickpea rhizosphere and coded as MD-11, MD-17, MD-23, MD-29, MD-42, MD-47, MD-51, MD-55, MD-60, MD-62, MD-68 and MD-70. Each isolate exhibited distinct colony morphology and shape on ZSAM agar plates.

### 3.2 Zn-solubilization potential: Zn solubilization efficiency (ZSE) and Zn-solubilization index (ZSI) and Zn quantification

**Figure 1:**
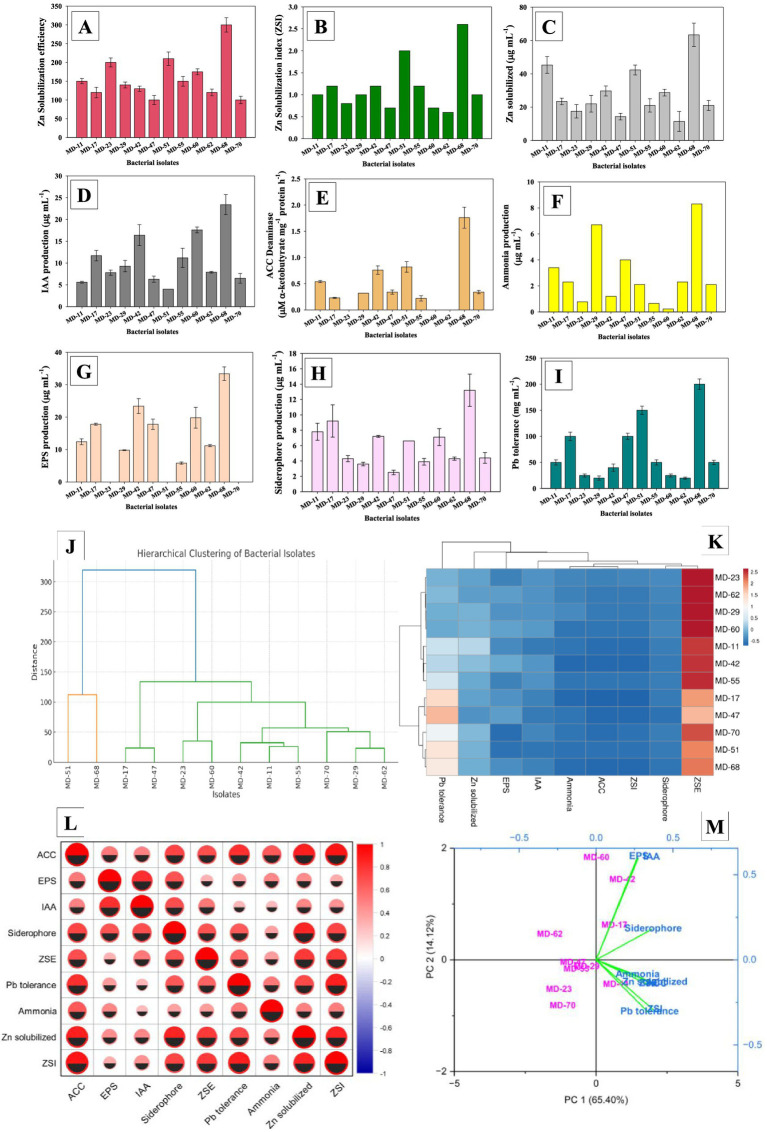
Screening of bacterial isolates for Zn solubilization efficiency **(A)**, Zn solubilization index **(B)**, Zn quantification **(C)** and growth-promoting traits; IAA production **(D)**, ACC Deaminase **(E)**, ammonia production **(F)**, EPS production **(G)**, siderophore production **(H)** and Pb tolerance **(I)**. Bar diagrams represent mean values of three replicates (*n* = 3). Mean values followed by the same letter are significantly different (*p* < 0.05) according to DMRT. The multivariate analysis reveals functional clustering of bacterial isolates; Hierarchical clustering **(J)**, heat map **(K)**, Pearson correlation **(L)** and PCA bio-plot **(M)**.

Bacterial isolates exhibited significant (*p* < 0.05) differences in Zn-solubilization index (ZSI) after 48 h and 72 h (ZSI; 72). Isolate MD-68 had highest ZSI values (2.6) followed by MD-51 (2.0) and MD-55 (1.2) (Figure 1A). Zinc solubilization efficiency (ZSE) likewise varied among isolates. Among all isolates, MD-68 possess greatest ZSE (300). Lowest ZSE (120) was recorded for isolate MD-17 (Figure 1B). The quantity of Zn solubilized differed among bacterial isolates grown in liquid medium supplemented with ZnO. Maximum Zn content (63.4 μg mL^˗1^) was recorded for isolate MD-68, which exhibited the highest solubilization efficiency on agar media. Isolate MD-51 solubilized 42.3 μg mL^˗1^ Zn, which was significantly (*p* < 0.05) lower than MD-68. The least effective isolate, MD-70, solubilized only 21 μg mL^˗1^ Zn. Notably, MD-68 enhanced Zn solubilization by approximately 202% compared to MD-70 and by 50% compared to MD-51 (Figure 1C). The varying abilities of the isolates to mobilize insoluble Zn are highlighted by the variations in ZSE, ZSI, and Zn solubilization content. In both agar and broth tests, isolate MD-68 consistently showed highest solubilization potential, demonstrating robust metabolic activity and effective organic acid secretion. Conversely, isolates with lower ZSE and Zn release likely possess fewer mechanisms for solubilization. These results support the contention that MD-68 is a viable candidate for development of biofertilizers to increase Zn bioavailability in crops.

### 3.3 Screening for growth-promoting activities of bacterial isolates

The isolates exhibited significant and variable production of plant growth promoting metabolites, including indole-3-acetic acid (IAA) (Figure 1D), 1-amino cyclopropane carboxylate (ACC) deaminase (Figure 1E), ammonia (Figure 1F), exopolysaccharide (EPS) (Figure 1G), and siderophore (Figure 1H). Among all isolates, MD-68 produced the highest level of IAA (23.4 μgIAAmL^˗1^), ACC Deaminase (1.78 μM *α*-ketobutyrate mg^−1^ protein h^−1^), EPS (33.5 μg mL^˗1^), and siderophores.

### 3.4 Lead tolerance among rhizobacteria

The recovered isolates exhibited a wide range of Pb tolerance, ranging from 20 to 200 mg mL ^˗1^. Isolate MD-68 showed the highest tolerance (200 mg mL^˗1^), followed by MD-51 (150 mg mL^˗1^) and MD-17/MD-47 (100 mg mL^˗1^ each). Isolates MD-11, MD-55, and MD-70 exhibited moderate tolerance (50 mg mL^˗1^ each), while MD-23 and MD-60 tolerated 25 mg mL^˗1^. The lowest tolerance was observed in MD-29 and MD-62 (20 mg mL^˗1^) (Figure 1I). Isolate MD-68 shows robust resistance and is a viable option for additional Pb bioremediation research as demonstrated by these results. Variations in Pb tolerance among isolates may reflect differences in metal efflux systems, biosorption capacities, and production of stress-protective metabolites (Ahemad, 2014). The diversity of tolerance levels emphasizes the particular value of highly tolerant strains like MD-68 for application in Pb-contaminated soils.

### 3.5 Multivariate analysis

Multivariate analysis revealed functional clustering among the bacterial isolates. Hierarchical clustering showed that bacterial isolates formed distinct groups based on their PGP and Pb tolerance traits, indicating functional similarity within clusters (Figure 1J). Considerable variation was observed among isolates in ACC deaminase, EPS, IAA, siderophore, Zn solubilization, Pb tolerance, and ammonia production. Some isolates, such as MD-23, MD-62, MD-29 exhibited strong expression of multiple traits, while others displayed lower activity levels (Figure 1K). Pearson correlation analysis indicated positive relationship among EPS, IAA, siderophore production, Zn solubilization, and metal stress tolerance, whereas, a few traits showed weak or negative correlations (Figure 1L). In PCA bio-plot, the first two principal components (PC1: 65.4%, PC2: 14.1%) explain ~80% of the total variance. Certain isolates, including MD-2, MD-60, MD-62, MD-70, were strongly associated with the key PGP traits and Pb tolerance, highlighting their potential as effective bioinoculants (Figure 1M).

### 3.6 Molecular identification of pb-tolerant MD-68

**Figure 2:**
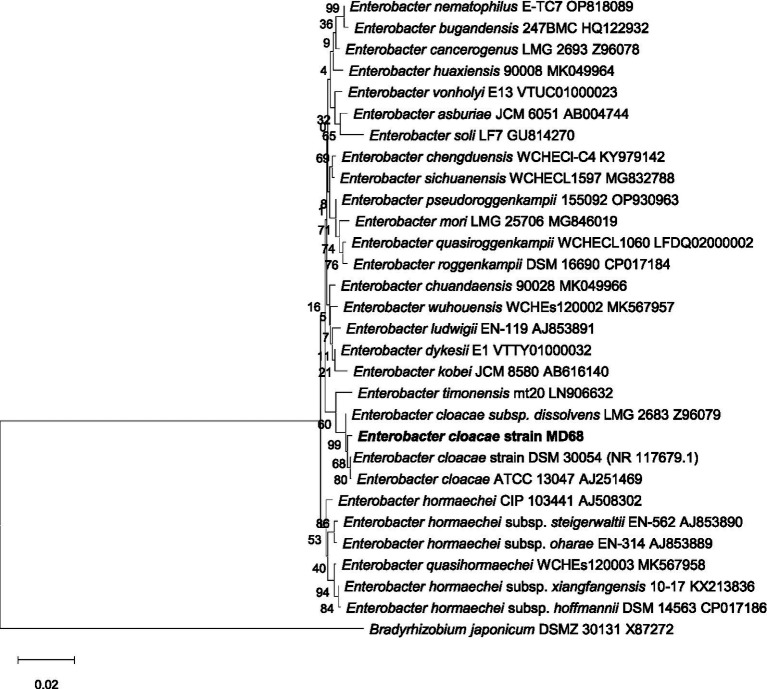
Phylogenetic tree of *E. cloacae* MD-68 and related bacterial strains retrieved from GenBank data constructed from MEGA 8.0.

The Pb-tolerant isolate MD-68 was molecularly identified using 16S rRNA gene sequencing. The amplified 16S rRNA fragment (~1.5 kb) was sequenced and analysed using BLASTn against the NCBI GenBank database. Sequence similarity analysis confirmed its taxonomic affiliation with *E. cloacae* DSM 30054 (Acc. No. NR 117679.1), showing a 99% identity. The obtained sequence was deposited in GenBank under accession number PV155251.1 (1,363 bp). Phylogenetic analysis further clustered isolate MD-68 with reference strains of *E. cloacae*, placing it firmly within the *E. cloacae* clade (Figure 2), placing it within the *E. cloacae* clade. These results confirm that the highly Pb-tolerant isolate MD-68 belongs to the genus *Enterobacter* and possesses close genetic relatedness to known metal-resistant strains. Its genetic identification reinforces its potential as an efficient Pb-bio-remediator, consistent with previous reports highlighting *Enterobacter* spp. for their metal resilience and key roles in plant–microbe–metal interactions.

### 3.7 Effect of pb on ZnS potential and growth-promoting traits of *E. cloacae* MD-68

**Figure 3:**
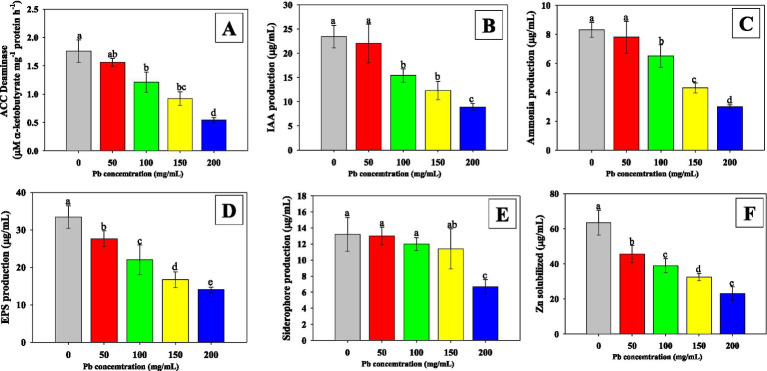
The effect of different Pb concentrations on growth-promoting substances of *E. cloacae* MD-68; ACC Deaminase **(A)**, IAA production **(B)**, Ammonia production **(C)**, EPS production **(D)**, siderophore **(E)** and Zn solubilization **(F)**. Bar diagrams represent mean values of three replicates (*n* = 3). Mean values followed by the same letter are significantly different (*p* < 0.05) according to DMRT.

The effect of increasing Pb concentration on the solubilization efficiency (ZSE), solubilization index (ZSI), and production of growth-promoting substances by *E. cloacae* MD-68 was evaluated. All PGP traits declined significantly with rising Pb levels, with the highest inhibition observed at 200 mg mL^−1^. At this concentration, ZSE (Figure 3) and ZSI of *E. cloacae* MD-68 decreased by 64.2 and 52.3%, respectively. Similarly, the synthesis of key metabolites was markedly reduced, including IAA (−34.5%), ACC deaminase (−45.6%), siderophores (−32.3%), and EPS (−45.6%) compared to the control. Although *E. cloacae* MD-68 retained measurable activity for all traits even under extreme Pb stress, these reductions indicate that high Pb levels adversely affect its metabolic capacity and PGP potential. Such inhibition likely results from oxidative damage, enzyme inactivation, and disruption of membrane integrity, as also reported for other PGPR exposed to heavy metals (Syed et al., 2021). Despite these challenges, *E. cloacae* MD-68 exhibited notable resilience, maintaining detectable levels of IAA, ACC deaminase, siderophore, and EPS production under severe Pb stress. This persistence of these functions suggests that *E. cloacae* MD-68 can mitigate Pb toxicity, enhance host plant stress tolerance, and improve nutrient availability. This adaptive tolerance aligns with reports that metal-tolerant PGPR retain PGP activity under stress, making them promising candidates for crop improvement and bioremediation in contaminated soils (Deepali et al., 2025). Overall, the ability of *E. cloacae* MD-68 to sustain vital PGP functions under elevated Pb conditions highlights its potential as an effective bioinoculant for use in Pb-polluted environments.

### 3.8 Effect of Zn, ZnO-NP and zinc solubilizing (ZS) *E. cloacae* on growth attributes of Pb-stressed chickpea

**Figure 4:**
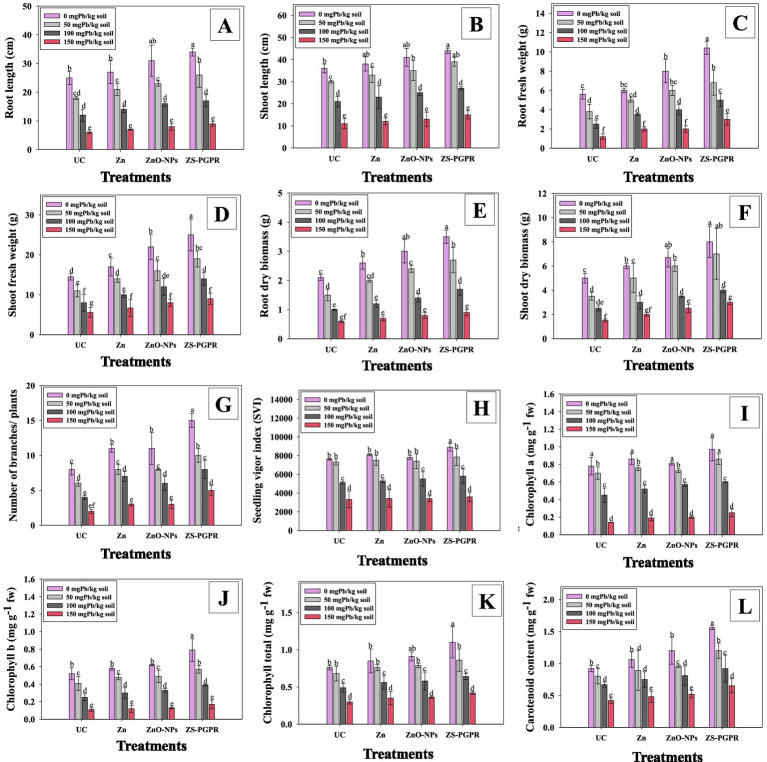
Effect of Zn fertilizer, ZnO-NPs and Zn-solubilizing *E. cloacae* (Zs-*Ec*) MD-68 on growth, biomass and leaf pigments of chickpea plants subjected to three concentrations (50, 100 and 150 mgPbkg^−1^ soil): root length **(A)**, shoot length **(B)**, root fresh weight **(C)** shoots fresh weight **(D)**, root biomass **(E)**, shoot biomass **(F)**, number of branches/plants **(G)**, seedling vigor index **(H)**, chlorophyll a **(I)**, chlorophyll b **(J)**, total chlorophyll **(K)** and carotenoid content **(L)**. Bar diagrams represent mean values of three replicates (*n* = 3). Mean values followed by the same letter are significantly different (*p* < 0.05) according to DMRT.

Chickpea plants exposed to increasing Pb concentrations showed a significant (*p* < 0.05) reduction in root lengths (RL) and shoot length (SL). At 150 mgkg^−1^ RL and SL decreased by 76% (Figure 4A) and 84% (Figure 4B) compared to the untreated control. However, supplementation with Zn and ZnO-NPs, and bacterial inoculation mitigated Pb-induced growth inhibition and promoted plant development. In 50 mg Pb kg^−1^ soil, these treatments increased RL by 14, 21.7, and 30.7%, and SL by 21.4, 31.2, and 42.1%, respectively, compared to the Pb-only treatment. Similarly, root fresh weight (RFW) and shoot fresh weight (SFW) declined significantly under Pb stress. At 100 and 150 mg Pb kg^−1^, RFW decreased by 55.3 and 78.5%, and SFW by 44.8 and 67.9%, respectively, relative to the control (Figures 4C,D). Application of Zn, ZnO-NPs, and Zns-*Ec* alleviated these negative effects. In 50 mg Pb kg^−1^ soil, RFW increased by 24, 36.6, and 44.4%, respectively, while improvements in SFW followed the order ZnO-NPs > Zns-*Ec* > Zn. The Pb toxicity is known to impair plant growth by disrupting photosynthesis, enzymatic activities, and nutrient uptake (Hadi and Aziz, 2015). The observed decline in RL and SL under Pb stress aligns with previous findings in chickpea and other crops (Hussain et al., 2013). Pb interferes with the absorption of essential nutrients such as calcium (Ca) and magnesium (Mg), resulting in oxidative stress, membrane damage, and nutrient imbalances (Barzin et al., 2025). Zn supplementation counteracted these effects by stabilizing cellular membranes, activating antioxidant defense systems, and improving nutrient uptake (Emamverdian et al., 2022). ZnO-NPs further enhanced these benefits due to their higher bioavailability and sustained Zn release, leading to improved antioxidant activity, root and shoot elongation, and Zn accumulation in Pb-stressed chickpea. Likewise, the Zn-solubilizing rhizobacterium Zns-*Ec* promoted growth under heavy metal stress through Zn solubilization, phytohormone (e.g., auxin) production, and reduction of Pb bioavailability in the rhizosphere. PGPR-mediated nutrient cycling and metal immobilization may also contribute to increased biomass accumulation (Grover et al., 2021). Similar synergistic effects of ZnO-NPs and metal-tolerant rhizobacteria on plant growth under Cd-contaminated conditions were reported in wheat (Sehrish et al., 2024a, 2024b).

### 3.9 Biomass, branching patterns, and seedling vigor index of pb-treated chickpea as influenced by Zn, ZnO-NPs, and Zns-*Ec*

Biomass, branch count, and seedling vigor index (SVI) varied in chickpeas treated with Pb, Zn and ZnO-NPs, and primed with the PGPR strain. Root dry biomass (RDB) (Figure 4E), shoot dry biomass (SDB) (Figure 4F), number of branches per plant (Figure 4G), and SVI (Figure 4H) all significantly (*p* < 0.05) decreased as Pb dosage increased from 50 to 150 mgkg^−1^. For example, at 50, 100, and 150 mgkg^−1^ soil, Pb significantly reduced RDB (23.4, 52.3, and 71.4%), SDB (19.8, 43.6 and 65.4%) and SVI (23.3, 38.9 and 65.7%) compared to control. According to Kanwal et al. (2020), the observed decreases in biomass and SVI correspond with results of other studies that document the inhibitory effects of Pb on root and shoot development. Such growth impairment is probably caused by cellular damage, oxidative stress, and nutritional imbalances brought on by Pb. Application of Zn/ZnO-NPs and soil inoculation of metal-tolerant PGPR strain to Pb-stressed chickpea alleviated Pb toxicity and resulted in improved biomass and SVI under Pb stress. When plants were doped with 50 mgPbkg^−1^, application of Zn, ZnO-NPs, and Zns-*Ec* increased RDB (20.8, 29.9 and 44.5%,) SDB (17.8, 28.7, and 39.5%) and SVI (26.7, 47.8, and 62.4%) in comparison to the treatment with 50 mgPbkg^−1^ alone., The Zn, ZnO-NP, and Zns-*Ec* treatments improved plant growth parameters under Pb stress; however, effects differed somewhat, with PGPR imparting the greatest impact. This effect may be due to the dual function of PGPR, which alleviates metal stress through nutrient mobilization and also by promoting plant growth directly via phytohormone production and microbial interaction (Ranjan et al., 2024). Zinc and ZnO-NPs mitigate Pb toxicity primarily via nutrient supplementation and antioxidant protection, which may explain their lesser impact compared to PGPR. Zinc preserves membrane integrity and enzyme activity. ZnO-NPs improve nutrient bioavailability and stress tolerance, and metal-tolerant ZnS-PGPR regulated root architecture, phytohormone levels, and antioxidant defense (Muhammad et al., 2022). PGPR treatment resulted in the greatest impact on chickpea, demonstrating its ability to reduce Pb toxicity directly through metal immobilization, growth-promoting metabolite secretion, and enhanced nutrient uptake. These results highlight the contention that Zn fertilizers, ZnO-NPs, and Zns-*Ec* can work together to enhance plant growth under Pb stress, providing avenues for long-term crop management in contaminated soils.

### 3.10 Leaf pigments in pb-tressed chickpea as influenced by Zn, ZnO-NPs and ZS-*E. cloacae*

Increasing Pb concentration markedly reduced leaf pigments (chlorophyll and carotenoids) in chickpea. The 150 mgPbkg^−1^ treatment caused severe toxicity, leading to a greater reduction in chlorophyll *a* (Figure 4I), chlorophyll *b* (Figure 4J), total chlorophyll (Figure 4K), and carotenoids (Figure 4L) by 67.8, 73.5, 64.8, and 53.5%, respectively, over the control treatment. These results align with earlier findings that metal toxicity, particularly Pb, disrupts photosynthesis by inducing oxidative stress, impairing chlorophyll biosynthesis, and damaging photosystem II efficiency (Shu et al., 2012; Cunha et al., 2020). The decline in leaf pigments and photosynthetic efficiency under Pb stress reflects damage to chloroplast membranes and inhibition of pigment stability, as also reported by Gomes et al. (2014). Conversely, Zn fertilizer, ZnO-NPs, and Pb-tolerant Zns-*Ec* inoculation significantly improved pigment content under both normal and Pb-stressed conditions. In plants exposed to 50 mg Pb kg^−1^, Zn, ZnO-NPs, and Zns-*Ec* increased chlorophyll a by 21.2, 26.5, and 39.8%; chlorophyll b by 23.5, 28.0, and 48.7%; total chlorophyll by 19.6, 23.4, and 42.8%; and carotenoids by 26.7, 29.7, and 45.7%, respectively, compared with Pb-only treatments. These increases indicate that the photosynthetic machinery was effectively protected and optimized by the treatments. Zn plays a key role in stabilizing chloroplast membranes and activating antioxidant enzymes, thereby maintaining chlorophyll integrity (Sehrish et al., 2024a, 2024b). ZnO-NPs further enhance Zn bioavailability and protect against oxidative damage, leading to improved photosynthetic performance under metal stress (Kadri et al., 2024). In addition, Pb-tolerant PGPR such as Zns-*Ec* enhance nutrient uptake, promote chlorophyll synthesis, and produce phytohormones (e.g., auxins) that stimulate photosynthetic efficiency and mitigate metal toxicity (Pal et al., 2024). Among all treatments, Zns-*Ec* exerted the strongest positive effects on leaf pigments, likely due to its combined roles in nutrient solubilization, antioxidant enhancement, and phytohormone production.

### 3.11 Photosynthetic attributes of pb-stressed chickpea treated with Zn, ZnO-NP and Zns-*Ec*

**Figure 5:**
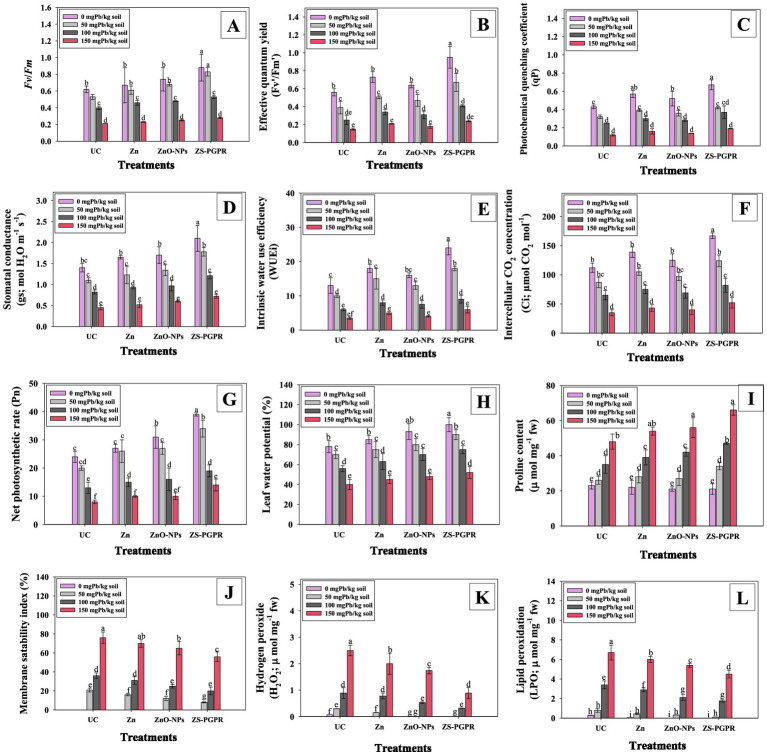
Effect of Zn, ZnO-NPs and Zs-*Ec* on chlorophyll fluorescence, gas exchange attributes, and oxidative stress parameters in Pb-stressed chickpea: Fv/Fm **(A)**, effective quantum yield **(B)**, photochemical quenching coefficient **(C)**, stomatal conductance **(D)**, intrinsic water use efficiency **(E)**, intercellular CO2 concentrations **(F)**, net photosynthetic rate **(G)**, leaf water potential **(H)**, proline content **(I)**, membrane stability index **(J)**, hydrogen peroxide content **(K)** and lipid peroxidation **(L)**. Bar diagrams represent mean values of three replicates (*n* = 3). Corresponding error bars represent standard deviation (S.D.). Mean values followed by the same letter are significantly different (*p* < 0.05) according to DMRT.

Photosynthetic attributes including maximum PSII efficiency (Fv/Fm) (Figure 5A), effective quantum yield (Fv’/Fm′) (Figure 5B) and photochemical quenching coefficient (qP) (Figure 5C) declined progressively with increasing Pb concentrations. At 150 mgPbkg^−1^, Fv/Fm, Fv’/Fm′, and qP decreased by 67.2, 54.7, and 58.9%, respectively, compared to the control. These reductions demonstrate that Pb stress severely disrupts the photosynthetic machinery of chickpea. However, application of Zn fertilizer, ZnO-NPs and Zns-*Ec* substantially alleviated Pb-induced toxicity and improved photosynthetic efficiency. At 50 mgPbkg^−1^, Zn, Zn-NPs and Zns-*Ec* increased Fv/Fm (28.8, 31.2 and 48.9%), Fv’/Fm′ (21.8, 25 and 42.2%) and qP (20.5, 24.5, and 36%), over Pb treatments only. The observed decreases in Fv/Fm, Fv’/Fm′, and qP under high Pb stress reflect impaired excitation energy transfer and electron transport in PSII reaction centers, indicating that Pb toxicity disturbs chlorophyll fluorescence and reduces the efficiency of light energy conversion (Qin et al., 2023). In contrast, the mitigating effects of Zn, ZnO-NPs, and Zns-*Ec* highlight their protective roles in maintaining PSII integrity and photochemical performance. Several synergistic mechanisms likely explain these improvements: (i) Zn serves as a structural and catalytic cofactor for numerous enzymes and proteins, stabilizing chlorophyll–protein complexes and photosynthetic membranes (Yruela, 2013). (ii) ZnO-NPs, with their enhanced surface reactivity and controlled Zn release, provide a more bioavailable source of Zn, supporting chloroplast functionality and repair (Wang et al., 2016). (iii) The Zns-*Ec* strain enhances Zn solubilization and produces key plant growth-promoting metabolites—such as IAA, siderophores, and ACC deaminase—that alleviate oxidative stress, reduce ethylene accumulation, and improve stomatal regulation.

### 3.12 Pb-stressed chickpea gas exchange characteristics under Zn, ZnO-NPs, and Zns-*Ec*

Gas exchange parameters including stomatal conductance (gs), intrinsic water use efficiency (IWUE), intercellular CO_2_ concentrations (Ci), net photosynthetic rate (Pn) and leaf water potential (LWP) declined markedly under increased Pb concentrations. At 150 mgPbkg^−1^, the reductions were most pronounced, with gs, IWUE, Ci, Pn, and LWP decreasing by 78.9, 66.7, 70.3, 65.7, and 60.8%, respectively, compared to the control (Figures 5D–H) compared to control. These declines indicates that both stomatal and non-stomatal processes are strongly inhibited by Pb toxicity. Lower gs and Ci suggest stomatal closure and restricted CO₂ diffusion into mesophyll cells, ultimately reducing Rubisco carboxylation efficiency (Flexas et al., 2016). The decreases in Pn and LWP further reflect damage to photosynthetic machinery and cellular membranes, leading to impaired water relations and CO₂ assimilation (Pottosin and Shabala, 2016). Application of Zn fertilizer, ZnO-NPs and Zns-*Ec* mitigated the adverse effects of Pb stress and significantly improved photosynthetic performance. Under 50 mgPbkg^−1^ treatment, Zn, Zn-NPs and Zns-*Ec* enhanced gs (18.8, 21.3 and 38.9%), IWUE (23.4, 29, and 50.1%) and Ci (25.6, 29.7, and 37.8%), Pn (18.9, 20 and 29.8%) and LWP (22.3, 26.7 and 44.5%), compared to the Pb-only treatment. The pronounced recovery in gas exchange traits under these treatments indicates improved water status and photosynthetic activity in Pb-stressed chickpea plants. Several mechanisms likely contribute to this mitigation: (i) Zn supplementation stabilizes chloroplast ultrastructure and maintains the activity of Zn-dependent enzymes such as carbonic anhydrase, enhancing photosynthetic CO₂ fixation (Fariduddin et al., 2021); (ii) ZnO-NPs provide a sustained release of bioavailable Zn, alleviating Pb-induced nutrient imbalance and supporting stomatal regulation (Raghib et al., 2020); and (iii) Zns-*Ec* enhances root function and nutrient uptake while producing of phytohormones, ACC deaminase, and osmoprotectants that improve root hydraulic conductivity, reduce ethylene stress, and maintain water balance under metal toxicity (Ha-Tran et al., 2021).

### 3.13 Proline content and relative leaf water potential

Proline content in chickpea leaves increased significantly with increasing Pb concentration, reaching 11.9, 34.4, and 52% higher than the control in the 50, 100, and 150 mg Pb kg^−1^ treatments, respectively (Figure 5I). The application of Zn, ZnO-NPs and Zns-*Ec* further enhanced the proline levels in Pb-stressed chickpea. For example, in the 50 and 100 mgPbkg^−1^ treatments, proline increased by 14 and 19.3% with Zn, 15.6 and 22.1% with ZnO-NPs, and 35.6 and 42.3% with Zns-*Ec*, compared to untreated plants. Proline functions as an osmotic regulator and antioxidant, accumulating in response to lead toxicity (Sofy et al., 2020), consistent with previous observations in wheat under Pb stress (Yang et al., 2011). The additional increase under Zn, ZnO-NPs, and Zns-*Ec* treatments is likely due to activation of antioxidant defenses and stabilization of cell membranes (Sehrish et al., 2024a, 2024b).

Conversely, relative leaf water (RLW) decreased with increasing Pb levels, reaching a minimum of 78.6% of control at 150 mg Pb kg^−1^. The Zn, ZnO-NPs, and Zns-*Ec* treatments improved RLW, with the greatest enhancement observed in plants inoculated with metal-tolerant PGPR. These treatments likely improved RLW by enhancing root function and water uptake, while Pb stress normally disrupts water absorption. PGPR-mediated production of phytohormones promotes root growth and water retention, and Zns-*Ec* additionally aids in water balance by reducing Pb bioavailability in the rhizosphere (Abdollahi et al., 2022). Overall, these results highlight the potential of Zn, ZnO-NPs, and Zns-*Ec* to improve water relations, increase proline accumulation, and promote growth in chickpea under heavy metal stress, supporting their use in sustainable agriculture.

### 3.14 Zn, ZnO-NPs, and Zns-*Ec* interact to lessen pb-induced oxidative stress in chickpea

Lipid peroxidation (LPO), hydrogen peroxide (H_2_O_2_), and membrane stability index (MSI).

Electrolyte leakage (EL) in Pb-doped and Zn, ZnO-NPs, and Zns-*Ec* -applied leaves was measured in order to determine the membrane integrity/stability index (MSI) of Pb-stressed chickpea. Regardless of Zn treatment, low Pb doses resulted in a slight or marginal increase in levels of EL and hydrogen peroxide (H_2_O_2_) in leaf tissue. High Pb dosages resulted in a significant (*p* ≤ 0.05) increase in oxidative stress. For instance, Pb concentrations of 100 and 200 mg kg^−1^ resulted in an increase in EL by 53.4 and 79.8%, respectively (Figure 5J), and H_2_O_2_ content by 54.5 and 82.3% (Figure 5K), respectively, compared to the untreated control. In contrast, exogenous Zn and ZnO-NP application and bacterial (Zns-*Ec*) inoculation significantly decreased oxidative stress and improved plant growth in Pb-treated chickpea by enhancing antioxidant defense, reducing cellular damage, and fostering general physiological stability. For example, Zn, ZnO-NPs and metal-tolerant Zns-*Ec* strain application significantly lowered EL (18.9, 27.8 and 40.4%) and H_2_O_2_ content (23.2, 25.6 and 34.5%) in chickpea leaves when applied to plants grown in the low Pb treatment (50 mgkg^−1^). A similar trend was observed with lipid peroxidation (LPO): the 50, 100 and 150 mgkg^−1^ Pb doses significantly increased LPO levels by 30.2, 45.6, and 55.9% respectively, over untreated control (Figure 5L). However, plant stress biomarkers decreased with treatment by Zn, NPs, and bacterial priming. Chickpea treated with 100 mgPbkg^−1^ had lower levels of LPO in leaf tissue in the order: Zns-*Ec* (49.8%) > ZnO-NPs (33%) > Zn (17.8%). The lower levels of EL, H2O2 and LPO in Zn, ZnO-NP and Zns-*Ec* -inoculated chickpea suggest that the interaction alleviates oxidative damage induced by ROS production under Pb stress.

### 3.15 Treatment with Zn, ZnO-NPs, and metal-tolerant Zns-*Ec* improved antioxidant defense responses in Pb-stressed chickpea

**Figure 6:**
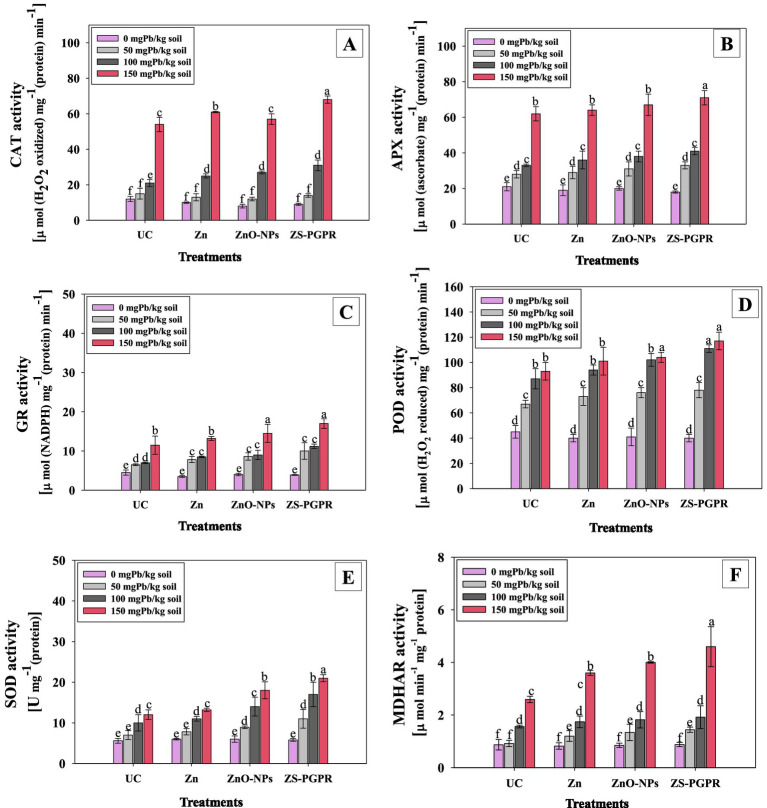
Antioxidant enzyme activity of chickpea plants in the presence of Pb, Zn fertilizer, ZnO-NPs and Zn-solubilizing PGPR: CAT **(A)**, APX **(B)**, GR **(C)** POD **(D)**, SOD **(E)** and MDHAR **(F)**. Bar diagrams represent mean values of three replicates (*n* = 3). Corresponding error bars represent standard deviation (S.D.). Mean values followed by the same letter are significantly different (*p* < 0.05) according to DMRT.

Antioxidant enzyme activity in chickpea leaves increased progressively with increasing Pb concentrations. In the absence of Pb, activities of glutathione reductase (GR), peroxidase (POD), catalase (CAT), ascorbate peroxidase (APX), superoxide dismutase (SOD), monodehydroascorbate reductase (MDHAR) remained stable. At low Pb doses, enzyme activities increased slightly, while higher doses (100 and 150 mgPbkg^−1^) caused marked elevations. In the 150 mgPbkg^−1^ treatment, CAT, APX, GR, POD, SOD, and MDHAR activities increased significantly by 77.7, 66.2, 63.6, 51.6, 59, and 73%, respectively, compared to the control (Figures 6A–F). This rise reflects Pb-induced oxidative stress, which stimulates antioxidant defenses to mitigate reactive oxygen species (ROS)-mediated damage (Huang et al., 2017; Rani et al., 2024). Application of Zn, ZnO-NPs, and Zns-Ec further enhanced antioxidant enzyme activity in Pb-stressed plants. At 150 mg Pb kg^−1^, treatment with Zn, ZnO-NPs, and Zns-Ec significantly increased CAT by 33.4, 40.7, and 51.2%; APX by 24.5, 29.8, and 41.2%; and GR by 20.7, 33.1, and 48.9%, respectively, relative to untreated plants. POD and SOD followed a similar trend, with increases of 18.9, 23.4, and 42% (POD) and 18.8, 22.3, and 33.2% (SOD). These enhancements indicate a stronger antioxidant defense under combined Zn and microbial treatments, consistent with reports that Zn-based amendments and metal-tolerant PGPR strains enhance enzymatic defense under heavy metal stress (Shah et al., 2021; Faizan et al., 2021; El-Ballat et al., 2023). Zinc stabilizes cell membranes and activates antioxidant systems (Rajput et al., 2021), while ZnO-NPs improve oxidative stress tolerance across crops (Faizan et al., 2021). Similarly, PGPR inoculation enhances antioxidant activity by promoting root development and overall plant growth, reducing the adverse effects of metal stress (El-Ballat et al., 2023). These findings highlight that Zn amendments and metal-tolerant microbial inoculation significantly strengthen plant defense mechanisms against Pb-induced oxidative stress (Faizan et al., 2021; Sarwar et al., 2023).

The interaction between Zn and Pb in plants is primarily antagonistic, where Zn competes with Pb for uptake and binding sites in roots due to their similar ionic radii and chemical behavior (Musielińska et al., 2016). Adequate Zn supply can thus reduce Pb absorption and translocation to shoots by competing for transporters and adsorption sites on root cell walls (Gupta et al., 2016). Moreover, Zn plays a structural and catalytic role in many antioxidant enzymes such as SOD and POD, thereby enhancing the plant’s ability to detoxify ROS generated under Pb stress (Priyanka et al., 2021). Zn also stabilizes membrane integrity and protein structure, mitigating Pb-induced LPO and enzyme inactivation. Hence, observed improvement in antioxidant activity and lower Pb accumulation in Zn- and ZnO-NP-treated plants can be attributed to synergistic effects of Zn-mediated defense reinforcement and Pb exclusion at the uptake level.

### 3.16 Pb accumulation, tolerance index, and phytotoxicity in chickpea under Zn, ZnO-NP, and Zns-*Ec* treatments

**Figure 7:**
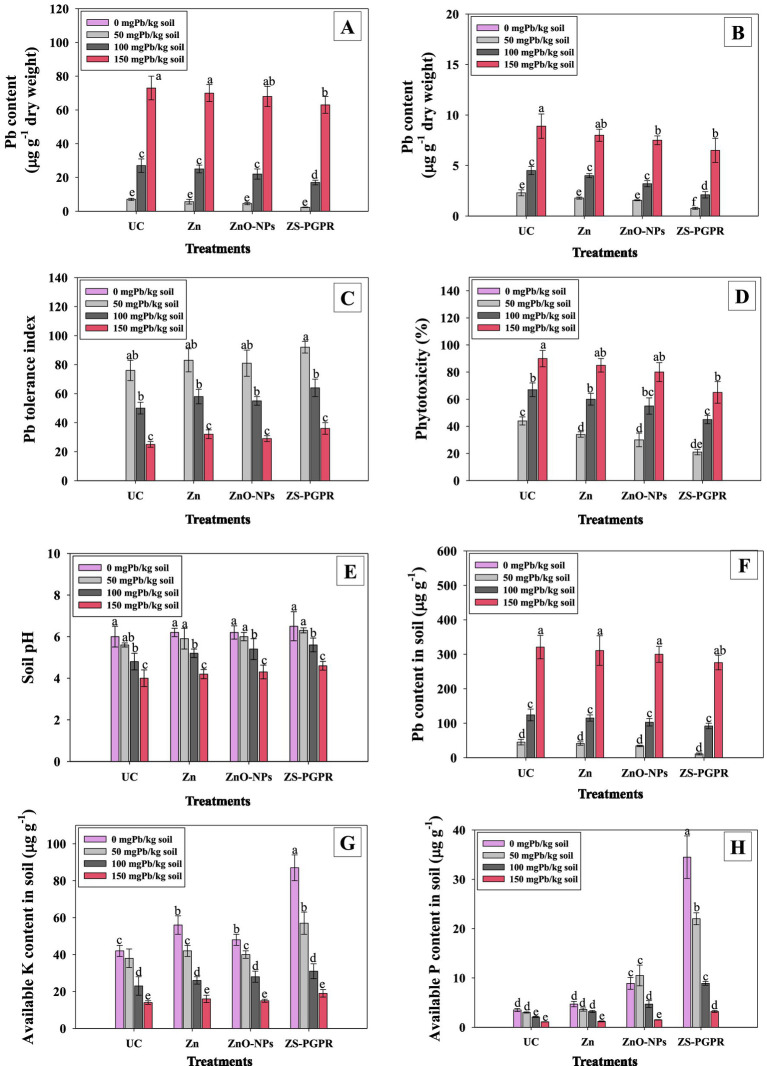
Effect of Zn and ZnO-NPs application, and ZnS-Ec inoculation on metal uptake and pH and nutrient content of rhizosphere soil of Pb-stressed chickpea plants: Pb content in roots **(A)**, Pb content in shoots **(B)**, Pb tolerance index **(C)**, percent phytotoxicity **(D)**, soil pH **(E)**, Pb content in soil **(F)** available P **(G)** and K content **(H)**. Bar diagrams represent mean values of three replicates (n = 3). Corresponding error bars represent standard deviation (S. D.). Mean values followed by the same letter are significantly different (p < 0.05) according to DMRT.

Lead uptake varied significantly in chickpea exposed to Pb and treated with Zn, ZnO-NPs, and Zns-*Ec*. At low Pb concentrations, uptake was limited, with root Pb content measuring 5.6, 14.5, and 34.5 μg Pb g^−1^ in soils containing 50, 100, and 150 mg Pb kg^−1^, respectively (Figure 7A). Most of the metal was sequestered in roots, with minimal translocation to shoots (Figure 7B). Higher soil Pb levels increased accumulation in both roots and shoots. These results align with previous studies showing that plants preferentially retain heavy metals in roots to protect aerial tissues from toxicity. Application of Zn and ZnO-NPs, and Zns-*Ec* significantly (*p* ≤ 0.05) reduced Pb uptake in root tissues by 20.1, 35.7, and 67%, respectively, and in shoots by 17.6, 29.5, and 61% in plants grown in soil with 50 mgPbkg^−1^. Additionally, these amendments improved the Pb tolerance index (Figure 7C) and reduced phytotoxicity (Figure 7D). The decrease in Pb uptake suggests that Zn, ZnO-NPs, and Zns-Ec enhance metal exclusion mechanisms or promote Pb detoxification (Ahmad and Ahmad, 2024). Zinc is known to stabilize cell membranes and support antioxidant defenses, enhancing plant resistance to metal stress, while ZnO-NPs, due to their high surface area and bioavailability, are effective in reducing heavy metal uptake (Umair Hassan et al., 2024). Zn and ZnO-NPs likely improve Pb exclusion at the root surface and facilitate sequestration in roots, limiting shoot translocation (Jafir et al., 2024). Metal-tolerant Zns-Ec mitigates Pb stress by producing exopolysaccharides that bind heavy metals and reduce their bioavailability (Ghosh et al., 2024). Moreover, by enhancing nutrient uptake and producing growth-promoting compounds, including phytohormones, PGPR strains modulate plant physiology, thereby supporting metal detoxification and promoting overall growth under stress conditions (Nazli et al., 2020).

### 3.17 Analysing physicochemical properties of rhizosphere soil after chickpea harvest

#### 3.17.1 Impact of Zn, ZnO-NPs, and Zns-*Ec* on soil pH and soil Pb content

Soil pH at the beginning of the study was 6.3, which declined progressively with increasing Pb doses, reaching its lowest value of 4.9 in the 150 mg Pb kg^−1^ treatment—a 36.5% reduction compared to the control (Figure 7E). Application of Zn, ZnO-NP and Zns-*Ec* significantly improved soil pH and reduced Pb content in the rhizosphere by 17.8, 20, and 27.8%, respectively, relative to control (Figure 7F). In trace metal-contaminated soils, pH reflects proton activity, and changes in soil chemistry under different pollution levels (Uddh-Söderberg et al., 2024). Zn ions and Zns-*Ec* enhance metal mobility, increasing Zn bioavailability and promoting root uptake (Shafigh et al., 2019). Notably, Zns-Ec-inoculated soil maintained higher pH than Pb-only-contaminated soil, indicating the beneficial impact of microbial inoculation. Metal-tolerant Zns-*Ec* can biologically mitigate Pb toxicity, likely by modulating soil chemistry and influencing enzymatic activity in plants (Patil et al., 2024). Acidification of the rhizosphere was indicated by a significant decrease in pH caused by Pb stress, which peaked in the 150 mg Pb kg^−1^ treatment. Overall, amendments with Zn, ZnO-NPs, and Zns-Ec effectively alleviated lead stress and improved rhizosphere health (Uddh-Söderberg et al., 2024; Shafigh et al., 2019).

### 3.18 Effect of Zn, ZnO-NPs, and Zns-*Ec* on available phosphorous and potassium in Pb-contaminated rhizosphere soil

Application of Zn fertilizer and ZnO NPs, and bacterial inoculation significantly improved the levels of available phosphorous (P) and potassium (K) in Pb-contaminated chickpea rhizosphere soil. Treatments with Zn, ZnO-NPs, and Zns-Ec increased P content by 18.2 24.5 and 29.5%, respectively, compared to control (Figure 7G). Similarly, in the 50 mgPbkg^−1^ treatment, rhizosphere K content increased by 24, 21, and 34.5%, under Zn, ZnO-NPs, and Zns-Ec application, respectively, compared to Pb treatment alone (Figure 7H). Improvement in soil nutrient availability, suggests that metal-tolerant bacterial strain Zns-*Ec* enhance soil quality and may facilitate the transformation or immobilization of heavy metals, thereby promoting plant growth (Alves et al., 2022). Lead has a great propensity to combine with compounds in soil or form complexes, resulting in its retention. Lead exhibits a strong tendency to complex with soil constituents, resulting in its retention within the rhizosphere. The observed increase in Pb content in both root and shoot tissues with rising Pb levels indicates that PGPR inoculation and ZnO-NP application may influence Pb mobility and distribution within the plant–soil system under Pb stress.

### 3.19 Multivariate analysis (PCA, heat map and Pearson correlation)

**Figure 8:**
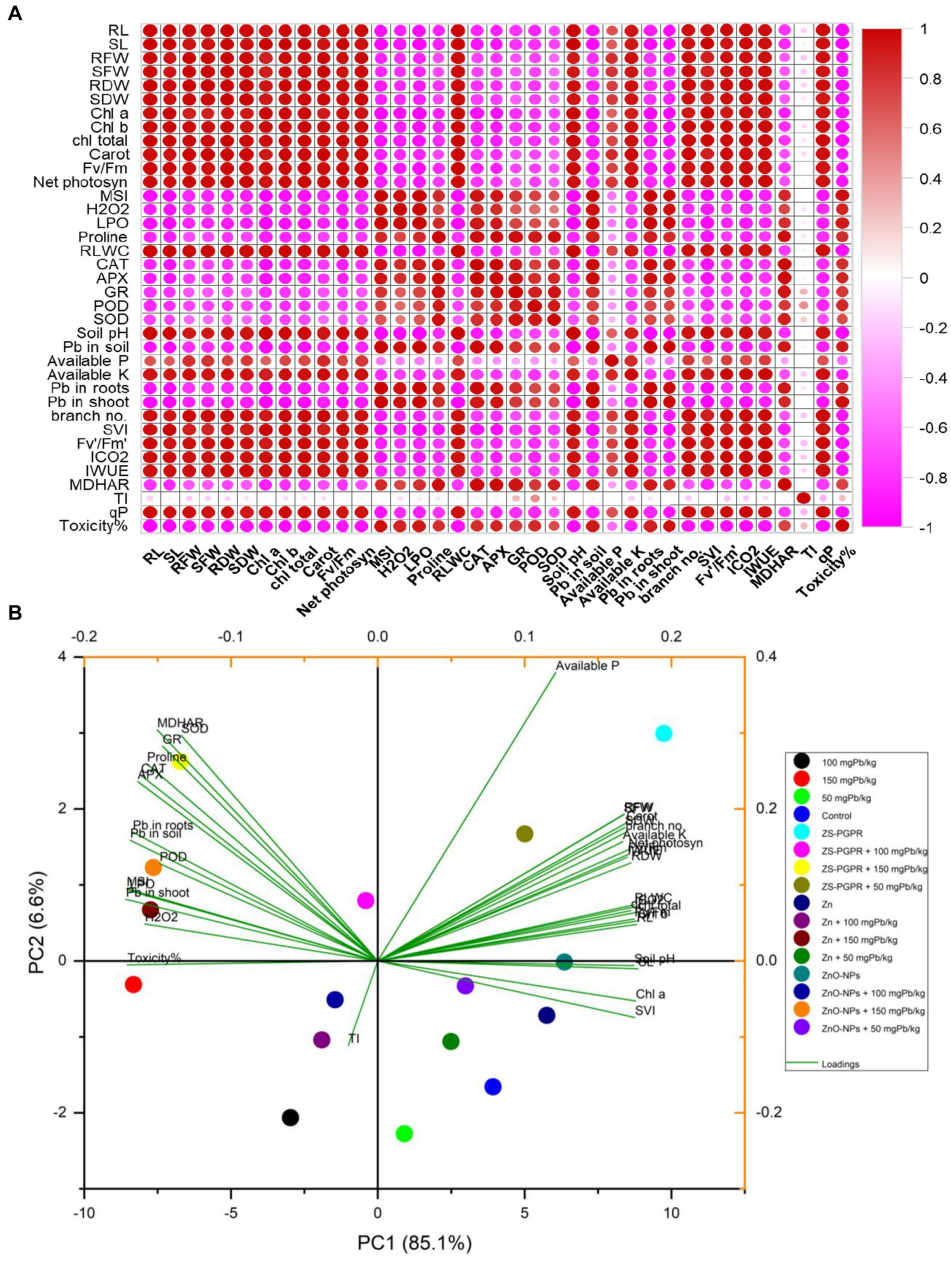
The correlation coefficient **(A)** and PCA bio-plot **(B)** figures showing the analyses of physio-biochemical, antioxidant and metal stress traits in chickpea plants under Pb-toxicity and applied/inoculated with Zn fertilizer, ZnO-NPs and Zn solubilizing *E. cloacae*. Here, RDW = root dry weight, RFW = root fresh weight, SDW = shoot dry weight, SFW = shoot fresh weight, LDW = leaf dry weight, LFW = leaf fresh weight, RL = root length, SL = shoot length, P = phosphorous, RWC = relative water content, N = nitrogen, CCI = chlorophyll color index, Pro = proline, SOD = superoxide dismutase, GR = glutathione reductase, Na = sodium, EL = electrolyte leakage, K = potassium, H2O2 = hydrogen peroxide, MDA = malondialdehyde, CAT = catalase, APX = ascorbate peroxidase, POD = peroxidase, carot = carotenoid, gs = stomatal conductance.

**Figure 9:**
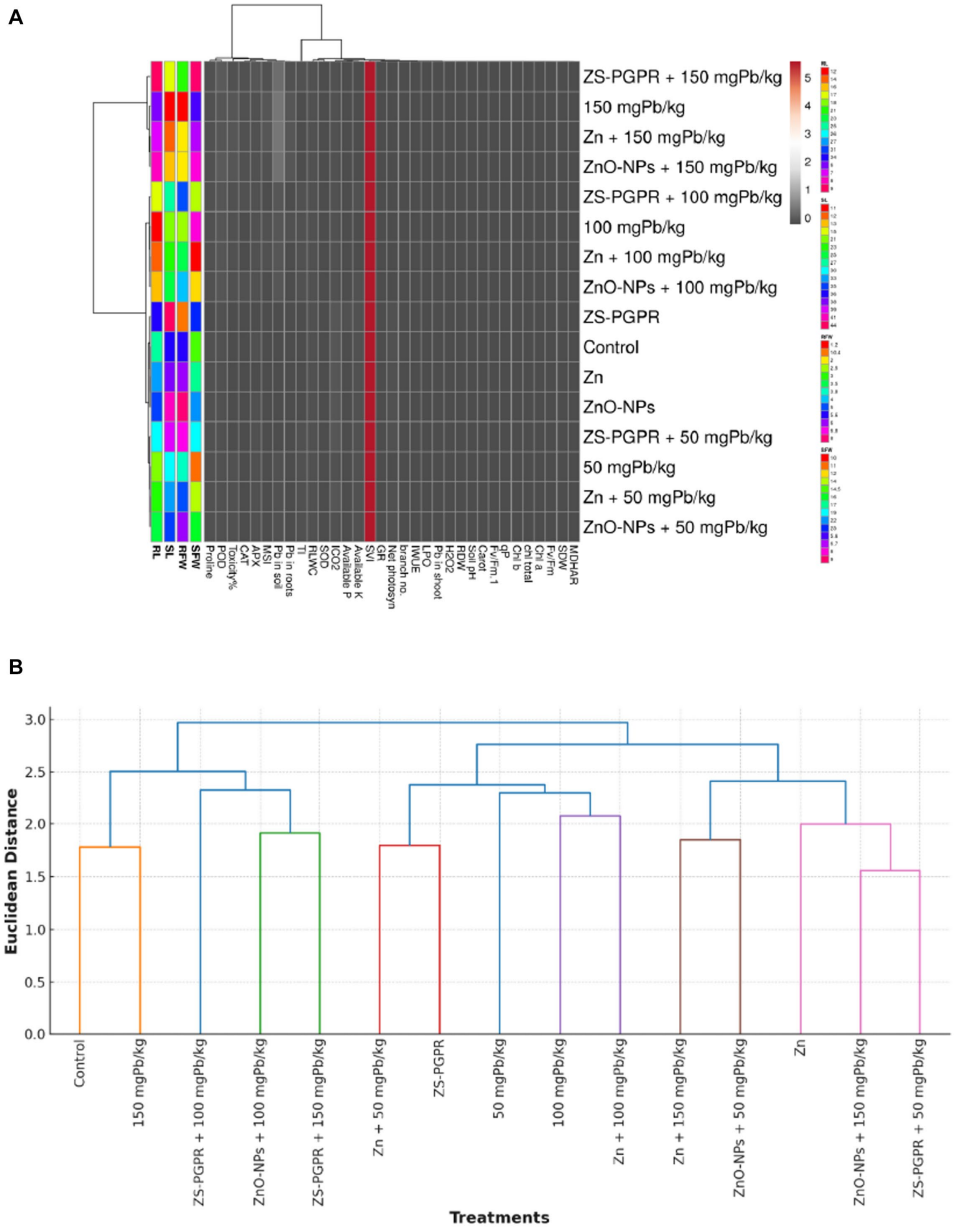
The heat map **(A)** and hierarchical clustering **(B)** showing the analyses of physio-biochemical, antioxidant and metal stress traits in chickpea plants under Pb-toxicity and applied/inoculated with Zn fertilizer, ZnO-NPs and ZsEc. Here, RDW = root dry weight, RFW = root fresh weight, SDW = shoot dry weight, SFW = shoot fresh weight, LDW = leaf dry weight, LFW = leaf fresh weight, RL = root length, SL = shoot length, P = phosphorous, RWC = relative water content, N = nitrogen, CCI = chlorophyll colour index, Pro = proline, SOD = superoxide dismutase, GR = glutathione reductase, Na = sodium, EL = electrolyte leakage, K = potassium, H2O2 = hydrogen peroxide, MDA = malondialdehyde, CAT = catalase, APX = ascorbate peroxidase, POD = peroxidase, carot = carotenoid, gs = stomatal conductance.

Multivariate analyses provided an integrated understanding of chickpea response to Pb stress and its mitigation by Zn, ZnO-NPs, and Zns-*Ec* treatments. Pearson correlation matrix revealed strong positive associations among growth traits (RL, SL, RFW, SFW, RDW, SDW), photosynthetic pigments (Chl a, Chl b, carotenoids), and physiological parameters (net photosynthesis, MSI, RWC, SVI, Fv/Fm, WUE). In contrast, oxidative stress indicators (H₂O₂, LPO, Pb accumulation) were negatively correlated with these traits but positively associated with antioxidant enzymes (CAT, APX, SOD, GR, MDHAR), indicating activation of defense response under Pb stress (Figures 8A,B). The heatmap corroborated these trends, showing that high Pb concentration (100–150 mgkg^−1^) markedly impaired growth, photosynthesis, and RWC while elevating oxidative stress. Conversely, Zn, ZnO-NPs, and particularly Zns-*Ec* treatments clustered closely with the control, reflecting restored physiological performance through enhanced photosynthetic efficiency, antioxidant activity, and nutrient availability. PCA further supported these patterns, explaining 91.56% of the total variation: Pb stress grouped with oxidative damage and Pb accumulation, whereas, Zns-*Ec*, Zn, and ZnO-NPs aligned with improved growth and physiological attributes (Figures 9A,B).

Conclusively, among the treatments, Zns-Ec MD-68 consistently showed the closest alignment with improved growth, physiological traits, and nutrient availability. This superior performance may be attributed to its multifaceted plant growth-promoting traits, including secretion of organic acids that enhance Zn solubilization and nutrient availability, siderophore production that chelates and immobilizes heavy metals, and intrinsic oxidative stress tolerance that supports root and shoot growth under Pb stress. Moreover, Zns-Ec may modulate phytohormone levels (e.g., auxins, gibberellins) and ACC deaminase activity, improving root architecture, water uptake, and overall plant resilience under metal toxicity (Grover et al., 2021; Arshad et al., 2023). These mechanisms likely explain the stronger mitigation of Pb toxicity and the enhanced plant performance observed with Zns-Ec inoculation compared to Zn and ZnO-NPs alone.

**Figure 10:**
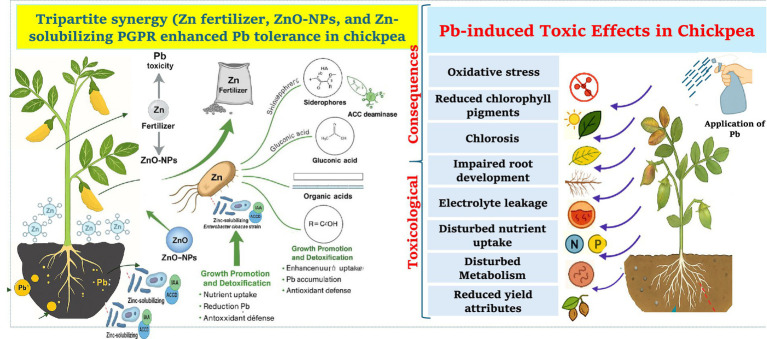
Toxic effect of Pb to chickpea plants, detoxification mechanisms and growth promotion via tripartite interaction utilization of Zn fertilizer, ZnO-NPs, and *E. cloacae*. Phytotoxic effects include oxidative stress, reduced chlorophyll content, stunted growth, and impaired root development. The application of Zn, ZnO-NPs, and *E. cloacae* synergistically alleviates these toxic effects. The PGPR strain contributes by producing growth-promoting biomolecules such IAA, siderophores, ACC deaminase, and EPS, along with Zn solubilization through production of gluconic acid and other organic acids. These mechanisms enhance nutrient uptake, reduce Pb accumulation, and stimulate antioxidant defense, resulting in improved plant growth and stress resilience.

To visually summarize the tripartite mechanism of Pb stress mitigation, we provide a schematic diagram (Figure 10) illustrating how Zn fertilizer, ZnO-NPs, and Zn-solubilizing, metal-tolerant *E. cloacae* (Zns-Ec) collectively enhance chickpea resilience. The figure depicts Pb-induced oxidative stress in roots and shoots, and shows how Zn and ZnO-NPs improve nutrient availability and photosynthesis, while Zns-Ec boosts antioxidant enzyme activity, reduces Pb uptake, and promotes plant growth through microbial-mediated mechanisms such as siderophore and organic acid production, ACC deaminase activity, and biofilm formation.

## 4 Conclusion

The present finding highlights a tripartite interaction between Zn amendments i. e. Zn fertilizer and ZnO-NPs, and Zn-solubilization *E. cloacae* (Zns-*Ec*), and chickpea under Pb stress. This synergistic combination significantly alleviated Pb toxicity by reducing Pb accumulation in roots and shoots and restoring photosynthetic efficiency and biomass production compared with Pb-stressed controls. The mitigation was closely associated with enhanced antioxidant defense (elevated SOD, CAT, APX, and GR activities) and improved nutrient homeostasis. These findings reveal a mechanistic link between Zn supplementation, microbial mediation, and oxidative stress alleviation—indicating that ZnO-NPs and Zns-Ec function complementarily to enhance Zn bioavailability and counteract Pb-induced damage. This study provides novel insight into Zn–microbe–plant synergy for heavy-metal detoxification and demonstrates its potential as a sustainable bio-nanotechnological approach for improving crop performance under contaminated conditions. Future studies should focus on field-level validation, nano–microbe formulation stability, and transcriptomic profiling to elucidate molecular pathways underlying this tripartite resilience mechanism.

## Data Availability

The datasets presented in this study can be found in online repositories. The names of the repository/repositories and accession number(s) can be found at: https://www.ncbi.nlm.nih.gov/genbank/, PV155251.1.
