# Supplementary material for: Zinc-based amendments and Zn-solubilizing beneficial bacteria mitigate lead-induced toxicity in chickpea (Cicer arietinum L.)
Source: Front Microbiol. 2025 Dec 12;16:1718122. doi: 10.3389/fmicb.2025.1718122 (PMC12741072; doi:10.3389/fmicb.2025.1718122)
Supplement: Supplementary file 1 [file Table_1.DOCX]

### **Indole-3-Acetic Acid (IAA) Production**

### The quantity of IAA produced by each bacterial isolate was estimated using the colorimetric method with Salkowski’s reagent, following a completely randomized design with three replications. Each isolate was inoculated into 25 mL of Luria Bertani (LB) broth supplemented with L-tryptophan (0.5–1 mg mL⁻¹) and incubated at 28 ± 2 °C for 48 h under shaking conditions. After incubation, cultures were centrifuged at 8000 rpm for 10 min, and 1 mL of the cell-free supernatant was mixed with 2 mL of Salkowski’s reagent. The mixture was kept in the dark for 30 min, and absorbance was recorded at 535 nm against an IAA standard curve (Batool et al., 2021).

**Exopolysaccharide (EPS) Production:**

Bacterial isolates were streaked on yeast extract–mannitol agar (YEMA) plates supplemented with 0.5% Congo red and incubated at 28 ± 2 °C for 3–5 days. Mucoid colony appearance indicated EPS production, which was further quantified by growing isolates in YEM broth (72 h, 28 °C, 120 rpm), followed by centrifugation (8000 rpm, 10 min). The supernatant was mixed with chilled ethanol (1:3 v/v) to precipitate EPS, which was oven-dried and weighed.

**Siderophore Production:**

Siderophore generation was evaluated using chrome azurol S (CAS) agar assay (Schwyn & Neilands, 1987). Bacterial isolates were spot-inoculated on CAS agar plates and incubated at 28 ± 2 °C for 48–72 h. The appearance of an orange/yellow halo zone around colonies indicated siderophore production. Siderophore units (%) were further quantified in CAS-shuttle solution at 630 nm.

**Ammonia Production:**

Ammonia production was tested by inoculating bacterial isolates into peptone water broth and incubating at 28 ± 2 °C for 48–72 h. After incubation, 1 mL of Nessler’s reagent was added to 1 mL of culture supernatant. The development of a brown to yellow coloration indicated ammonia production, and absorbance was measured at 450 nm for quantification.

**2.6 Molecular identification**

Genomic DNA of MD-68 strain was extracted using a commercial DNA extraction kit. The 16S rRNA gene was amplified by polymerase chain reaction (PCR) using universal primers 27F (5'-AGAGTTTGATCMTGGCTCAG-3') and 1492R (5'-TACGGYTACCTTGTTACGACTT-3'). PCR reactions were carried out in a 25 µL mixture containing 50 ng template DNA, 10 pmol of each primer, 200 µM dNTPs, 1.5 mM MgCl₂, 1× PCR buffer, and 1 U Taq DNA polymerase. Amplification was performed with the following conditions: initial denaturation at 95 °C for 5 min; 30 cycles of denaturation at 95 °C for 30 s, annealing at 55 °C for 30 s, extension at 72 °C for 90 s; and a final extension at 72 °C for 10 min. The amplified PCR products (~1.5 kb) were verified on 1% agarose gel electrophoresis, purified using a PCR purification kit, and sequenced bi-directionally. The obtained sequences were aligned and compared with reference sequences in the NCBI GenBank database using BLASTn. Phylogenetic analysis was performed with MEGA software using the Neighbor-Joining method.

**Table S1: Description of treatment plan**

| Treatment | Description |
| --- | --- |
| T1 | Control |
| T2 | Zn |
| T3 | ZnO-NPs |
| T4 | ZnS-*E. cloacae* MD-68 |
| T5 | 50 mgPbkg^-1^ soil |
| T6 | 100 mgPbkg^-1^ soil |
| T7 | 150 mgPbkg^-1^ soil |
| T8 | Zn + 50 mgPbkg^-1^ soil |
| T9 | Zn + 100 mgPbkg^-1^ soil |
| T10 | Zn + 150 mgPbkg^-1^ soil |
| T11 | ZnO-NPs + 50 mgPbkg^-1^ soil |
| T12 | ZnO-NPs + 100 mgPbkg^-1^ soil |
| T13 | ZnO-NPs + 150 mgPbkg^-1^ soil |
| T14 | MD-68 + 50 mgPbkg^-1^ soil |
| T15 | MD-68 + 100 mgPbkg^-1^ soil |
| T16 | MD-68 + 150 mgPbkg^-1^ soil |
